# A three-dimensional framework for acute appendicitis integrating etiology, pathological severity, and host response

**DOI:** 10.1097/MS9.0000000000005330

**Published:** 2026-07-21

**Authors:** Radomir Gelevski, Gjorgji Jota, Blagica Krsteska, Vladimir Andreevski, Mirjana Shosholcheva

**Affiliations:** a Department of Surgery, General Hospital Kumanovo, Kumanovo, North Macedonia; b University Clinic for Digestive Surgery, Ss Cyril and Methodius University, Skopje, North Macedonia; c Medical Faculty, Institute of Pathology, Ss Cyril and Methodius University, Skopje, North Macedonia; d University Clinic for Gastroenterohepatology, Ss Cyril and Methodius University, Skopje, North Macedonia; e Medical Faculty, Ss Cyril and Methodius University, Skopje, North Macedonia

**Keywords:** acute appendicitis, biomarkers, conceptual framework, disease classification, disease severity, host systemic response

## Abstract

Acute appendicitis is one of the most common causes of emergency abdominal surgery, yet clinicians continue to face difficulties in predicting disease severity, interpreting laboratory biomarkers, and explaining heterogeneous clinical presentations. Current classification systems primarily rely on severity-based progression models or etiology-based disease subtypes; however, both approaches incompletely explain the frequent discordance observed between pathological severity, systemic inflammatory response, microbiological findings, and clinical course. This conceptual limitation contributes to inconsistent biomarker performance and variability in study design across the appendicitis literature. In this conceptual review, we analyze the limitations of existing severity- and etiology-based frameworks and synthesize clinicopathological, microbiological, and biomarker evidence into an integrated multidimensional model of acute appendicitis. The proposed framework conceptualizes the disease along three interacting but partially independent axes: etiologic substrate, local pathological severity, and host systemic response. The etiologic substrate describes the initiating mechanism of appendiceal inflammation, local pathological severity reflects the extent of structural tissue injury, and host systemic response determines the magnitude of inflammatory amplification and biomarker expression. Recognizing host response as an independent modulating dimension provides a biologically plausible explanation for discordant biomarker findings and heterogeneous clinical trajectories. By reframing acute appendicitis as a multidimensional disease process rather than a binary entity or simple temporal continuum, this framework offers a coherent conceptual basis for improved interpretation of biomarkers, better study design, and more precise clinicopathological correlation. Adoption of such integrative models may ultimately support improved risk stratification and biologically informed management strategies in patients with acute appendicitis.

## Introduction

Acute appendicitis remains one of the most common indications for emergency abdominal surgery worldwide, yet its biological classification continues to be a subject of debate^[^1–7^]^. Traditionally, appendicitis has been described along a spectrum of severity, ranging from uncomplicated inflammation to gangrenous or perforated disease^[^1,4,8^]^. This severity-based framework has shaped clinical decision-making, surgical timing, and the interpretation of laboratory biomarkers^[^1,4^]^. However, accumulating clinical, microbiological, and pathological evidence suggests that appendicitis may not represent a single disease entity progressing uniformly over time, but rather a heterogeneous group of conditions with distinct underlying etiologies^[^2,3,6^]^.

HIGHLIGHTS

- Acute appendicitis shows discordance between pathology and systemic response.
- Severity and etiology models alone cannot explain disease heterogeneity.
- A three-dimensional framework integrates etiology, severity, and host response.
- Host systemic response explains variability in biomarker performance.
- Multidimensional classification may improve risk stratification and research design.

In parallel with severity-based models, etiology-driven concepts of appendicitis have emerged, proposing that uncomplicated and complicated forms may arise from fundamentally different pathogenic mechanisms^[^2,3,5^]^. These include luminal obstruction, primary bacterial invasion, polymicrobial virulence, ischemic injury, and host-specific inflammatory responses^[^5^]^. Such models help explain clinical observations that are difficult to reconcile within a simple temporal continuum, including early perforation, disproportionate systemic inflammation, and variability in microbiological profiles^[^3,6^]^. Nevertheless, etiology-focused frameworks often fail to account for the wide range of inflammatory severity observed within ostensibly similar pathological categories.

The coexistence of these two conceptual approaches – severity-based and etiology-based – has led to substantial heterogeneity in study design, patient stratification, and interpretation of results^[^1,2,7^]^. Biomarker research illustrates this tension particularly well: markers such as C-reactive protein, neutrophil-to-lymphocyte ratio, and hyperbilirubinemia are frequently evaluated as predictors of “complicated” appendicitis, yet their performance varies widely across studies^[^9–13^]^. This inconsistency may reflect not analytical failure, but conceptual misalignment – specifically, the conflation of disease severity with disease etiology.

Recent clinicopathological observations further challenge binary classification schemes. Localized but histologically severe inflammation may occur without systemic biochemical derangement^[^14,15^]^, while some patients exhibit early systemic responses despite limited local tissue damage^[^16,17^]^. Such findings suggest that severity and etiology are related but independent dimensions of appendiceal disease. Failure to distinguish between them risks oversimplification and may obscure meaningful biological signals.

Against this background, we propose that acute appendicitis should not be conceptualized solely as a binary condition or as a linear continuum of progression, but rather as a structured multidimensional framework in which etiologic substrate, local pathological severity, and host systemic response function as partially independent yet dynamically interacting dimensions. By explicitly separating initiating mechanisms from structural tissue injury and from systemic inflammatory amplification, this framework provides a coherent explanation for the discordance observed between pathology, biomarkers, microbiology, and clinical trajectories. Rather than replacing existing models, this approach integrates and extends them within a unified interpretative structure intended to improve conceptual clarity and research alignment^[^6,7^]^.

## Methods of literature selection and conceptual synthesis

This manuscript was developed as a narrative conceptual review and integrative synthesis of the contemporary literature on acute appendicitis. A structured literature search was performed using PubMed, Scopus, and Google Scholar to identify studies addressing appendicitis pathophysiology, disease classification, biomarkers, microbiology, and systemic inflammatory response.

The literature search focused primarily on publications from 2000 to 2026 and included combinations of the following keywords: “acute appendicitis,” “complicated appendicitis,” “uncomplicated appendicitis,” “biomarkers,” “hyperbilirubinemia,” “microbiome,” “pathophysiology,” “host response,” “systemic inflammation,” and “disease classification.” Additional relevant references were identified through citation tracking of key articles and reviews.

Studies were selected based on their relevance to conceptual models of appendicitis progression, etiologic mechanisms, clinicopathological correlation, microbiological findings, and interpretation of inflammatory biomarkers. Priority was given to studies providing insight into discordance between local pathological severity and systemic inflammatory response, as well as publications addressing heterogeneity in appendicitis presentation and outcomes.

Because the objective of the manuscript was conceptual integration rather than quantitative evidence synthesis, formal systematic review methodology and meta-analytic techniques were not applied. Instead, the selected literature was synthesized to construct a multidimensional interpretative framework integrating etiologic substrate, local pathological severity, and host systemic response.

## The severity-based (continuum) model of acute appendicitis

The traditional conceptualization of acute appendicitis is rooted in a severity-based continuum, in which the disease progresses through increasingly severe pathological stages over time^[^1,4,7,8^]^. Within this framework, appendicitis is viewed as a single disease entity that evolves from early mucosal inflammation to phlegmonous, gangrenous, and ultimately perforated forms if left untreated^[^18^]^. This model has been historically supported by surgical observations, pathological descriptions, and the temporal relationship between symptom duration and disease severity^[^16,17^]^.

The continuum model offers several practical advantages. It aligns with classical pathological grading systems^[^18,19^]^, provides a straightforward explanation for delayed perforation^[^16^]^, and supports the rationale for early surgical intervention to prevent progression^[^1,8^]^. Furthermore, it underpins many contemporary clinical scoring systems and treatment algorithms^[^7^]^, which implicitly assume that uncomplicated and complicated appendicitis represent different points along the same disease trajectory rather than biologically distinct processes.

However, the severity-based paradigm also has notable limitations. It presumes a relatively uniform starting point for all patients and implies that progression is primarily time-dependent^[^16,17^]^. This assumption fails to account for cases in which severe or perforated appendicitis develops early in the disease course^[^3,6^]^, as well as instances where prolonged symptoms are associated with only mild or localized inflammation^[^17^]^. Additionally, the continuum model does not adequately explain the marked interindividual variability in inflammatory response^[^9,10^]^, nor does it account for the influence of distinct etiologic drivers on disease behavior^[^2,3^]^.

As a result, while the severity-based model remains clinically intuitive and operationally useful, it provides an incomplete explanation for the heterogeneity observed in appendicitis presentations and outcomes.

## Biomarkers interpreted within the severity paradigm

Within the severity-based framework, laboratory biomarkers have been extensively investigated as surrogate indicators of disease progression and systemic inflammatory burden^[^4,9,10^]^. Commonly studied markers include white blood cell count, neutrophil-to-lymphocyte ratio, C-reactive protein, procalcitonin, and serum bilirubin^[^9–13^]^. These parameters are typically evaluated for their ability to discriminate between uncomplicated and complicated appendicitis, with the implicit assumption that higher values reflect more advanced stages along the severity continuum.

Among these biomarkers, inflammatory markers such as C-reactive protein and neutrophil-to-lymphocyte ratio generally correlate with the magnitude of systemic inflammatory response rather than the presence of specific pathological features^[^9,10^]^. Their diagnostic performance improves with increasing disease severity but remains limited in early or localized forms of appendicitis^[^9^]^. Procalcitonin, although more specific for systemic bacterial infection, often remains within normal ranges in localized inflammatory disease and therefore lacks sensitivity for early severe pathology^[^10^]^.

Serum bilirubin has emerged as a particularly illustrative example of the strengths and weaknesses of severity-based biomarker interpretation. Hyperbilirubinemia has been shown to demonstrate high specificity for advanced or complicated appendicitis, reflecting inflammation-induced cholestasis and endotoxemia^[^11–13^]^. However, its sensitivity remains low, and normal bilirubin levels are frequently observed in cases with significant local inflammation or even histologically severe disease^[^11,12^]^. This pattern suggests that bilirubin elevation is more closely linked to the systemic inflammatory response than to the extent of local tissue injury.

These observations highlight a critical limitation of interpreting biomarkers exclusively within a severity paradigm. Biomarkers predominantly reflect the host systemic response, although some parameters may also correlate indirectly with the extent of local tissue injury^[^2,3^]^.

## The etiology-based (dual-entity) model of acute appendicitis

In contrast to severity-based frameworks, etiology-based models conceptualize acute appendicitis as a heterogeneous group of biologically distinct disease processes rather than a single entity progressing along a uniform continuum^[^2,3,5,6^]^. Within this paradigm, uncomplicated and complicated appendicitis are viewed as arising from different pathogenic mechanisms, each with unique microbiological, inflammatory, and clinical characteristics.

Etiology-based models propose several primary pathogenic drivers, including luminal obstruction, primary bacterial invasion, polymicrobial virulence, ischemic injury, and host-specific immune modulation^[^5^]^. Obstruction-dominant mechanisms, often attributed to fecaliths or lymphoid hyperplasia, are thought to lead to gradual intraluminal pressure increase and localized inflammation, typically resulting in uncomplicated or slowly progressive disease^[^6,8^]^. In contrast, virulent polymicrobial infection or ischemic processes may precipitate rapid tissue necrosis and perforation, sometimes early in the disease course and with limited prodromal symptoms^[^6,19,20^]^.

This dual-entity perspective offers compelling explanations for clinical phenomena that challenge the continuum model, such as early perforation, disproportionate systemic inflammatory response, and inconsistent correlation between symptom duration and pathological severity^[^3,6,16,17^]^. Nevertheless, etiology-based models also have important limitations. They often imply rigid categorization of appendicitis into discrete disease entities, potentially overlooking the dynamic interaction between pathogenic triggers and host response^[^2,7^]^.

Etiologic classification alone does not reliably predict clinical severity, systemic inflammation, or postoperative outcomes, and substantial overlap exists between proposed etiologic categories^[^14,15^]^. Thus, while etiology-based frameworks contribute valuable insight into the biological diversity of acute appendicitis, they remain insufficient as standalone explanatory models.

## Pathological and clinical evidence of discordance between severity and etiology

Accumulating clinicopathological evidence demonstrates that disease severity and underlying etiology in acute appendicitis do not consistently align^[^14,15^]^. Numerous observations challenge the assumption that etiologic category reliably predicts inflammatory severity or that histopathological severity necessarily corresponds to systemic inflammatory response^[^9–13^]^.

From a pathological perspective, severe local inflammation may occur in the absence of systemic biochemical derangement. Transmural phlegmonous appendicitis with periappendiceal inflammatory extension and localized fibrinopurulent peritonitis can be observed without markers of systemic infection or cholestatic response^[^14,18,19^]^. Conversely, limited local tissue damage may coexist with pronounced systemic inflammation in certain patients^[^9–11^]^.

Clinical presentation further illustrates this dissociation. Symptom duration correlates imperfectly with pathological severity, and early perforation has been documented in patients with short clinical histories, while others exhibit prolonged symptoms without progression to complicated disease^[^16,17^]^. Such variability cannot be fully explained by either temporal progression alone or by fixed etiologic categories^[^2,3^]^.

Microbiological findings also fail to map neatly onto either severity- or etiology-based classifications. Polymicrobial flora are commonly identified across the full spectrum of appendicitis, and while certain organisms or bacterial profiles have been associated with complicated disease, substantial overlap exists^[^19–22^]^.

Incidental or secondary pathological conditions provide additional insight into this discordance. Appendiceal endometriosis, diverticula, or neoplasms may act as localized triggers for inflammation without necessarily inducing a systemic response^[^23,24^]^. In such cases, the etiologic substrate is identifiable, yet disease behavior remains confined, highlighting the independence of local causation from systemic severity.

Collectively, these lines of evidence indicate that neither severity-based nor etiology-based models alone adequately capture the complexity of acute appendicitis^[^6,7^]^.

## Microbiology in acute appendicitis: cause, modifier, or amplifier?

Microbiological factors occupy a central yet ambiguous position in contemporary models of acute appendicitis. Traditionally regarded as secondary to luminal obstruction and ischemia, bacterial involvement is now increasingly recognized as a potential driver of disease behavior^[^5,21^]^. However, the precise role of microbiology – whether causal, modifying, or amplifying – remains incompletely defined.

Culture-based and molecular analyses consistently demonstrate a polymicrobial environment within the inflamed appendix, typically dominated by anaerobic and facultative anaerobic organisms^[^19–22^]^. While certain bacterial species and microbial profiles have been associated with complicated appendicitis, similar organisms are frequently isolated in uncomplicated cases^[^19,21^]^. This overlap challenges attempts to define discrete microbiological signatures for different appendicitis phenotypes.

Rather than acting as a primary etiologic determinant in isolation, microbiological factors appear to modulate inflammatory intensity through bacterial load, virulence, and host–pathogen interactions^[^19,20,22^]^. Virulent polymicrobial communities may amplify local tissue damage and accelerate systemic inflammatory response, whereas lower bacterial burden or less aggressive microbial profiles may remain confined to localized inflammation^[^20,22^]^. In this context, microbiology functions less as a binary classifier and more as a severity modifier.

The inconsistency of microbiological findings across studies further reflects conceptual and methodological heterogeneity^[^21,22^]^. Differences in sampling techniques, timing of specimen collection, culture methods, and patient stratification contribute to variable results. Importantly, many studies categorize cases based on local pathological severity outcomes and then retrospectively infer microbiological causality, reinforcing circular reasoning^[^6^]^.

Taken together, current evidence supports a model in which microbiology may function both as an etiological contributor and as a modifier of inflammatory amplification, depending on biological context^[^6,20,22^]^.

## A hybrid framework integrating severity and etiology in acute appendicitis

The limitations of purely severity-based and etiology-based models suggest that neither framework alone can adequately explain the biological and clinical heterogeneity of acute appendicitis^[^1–3,6^]^. Instead, the available evidence supports a multidimensional view in which etiologic substrate, local pathological severity, and host systemic response represent distinct but dynamically interacting dimensions of disease expression.

Within this framework, the etiologic substrate axis addresses the biological mechanism initiating appendiceal inflammation, the local pathological severity axis defines the extent of structural tissue injury and peritoneal involvement, and the host systemic response axis reflects the degree to which the inflammatory process is systemically amplified or contained by the host. Although these dimensions are presented as analytically distinct, they remain biologically interconnected in clinical disease expression. Certain factors may influence more than one dimension simultaneously. For example, virulent polymicrobial infection may function both as an initiating etiologic driver and as an amplifier of systemic inflammatory response, while inflammatory biomarkers may partially correlate with tissue injury despite predominantly reflecting host-response intensity. The purpose of separating these dimensions is therefore not to imply complete biological independence but to provide conceptual clarity regarding the primary domain represented by specific clinical, pathological, microbiological, and biochemical findings.

In this structured model, the etiologic substrate encompasses mechanisms such as luminal obstruction, localized inflammatory triggers, polymicrobial virulence, ischemic injury, or incidental pathological conditions^[^5,23,24^]^. Local pathological severity ranges from mucosal inflammation to transmural necrosis, gangrene, perforation, and peritoneal extension, whereas the host systemic response reflects the magnitude of inflammatory and biochemical amplification generated by the local disease process. Recognizing these dimensions as partially independent but interacting domains helps reconcile discordant clinicopathological observations that cannot be adequately explained within single-axis models.

Importantly, local pathological severity should not be conflated with systemic inflammatory expression. Clinical biomarkers frequently reflect the host's systemic response rather than the extent of tissue injury itself. Recognizing this distinction allows for clearer interpretation of discordant findings, such as histologically advanced appendicitis occurring in the absence of marked systemic biomarker elevation^[^11,14,15^]^, or early systemic inflammatory response in patients with relatively limited structural damage^[^9,16,17^]^.

This hybrid two-axis structure provides a necessary foundation for understanding disease heterogeneity but remains incomplete without the consideration of host-specific inflammatory amplification, which is addressed in the following section.

**Figure 1.**
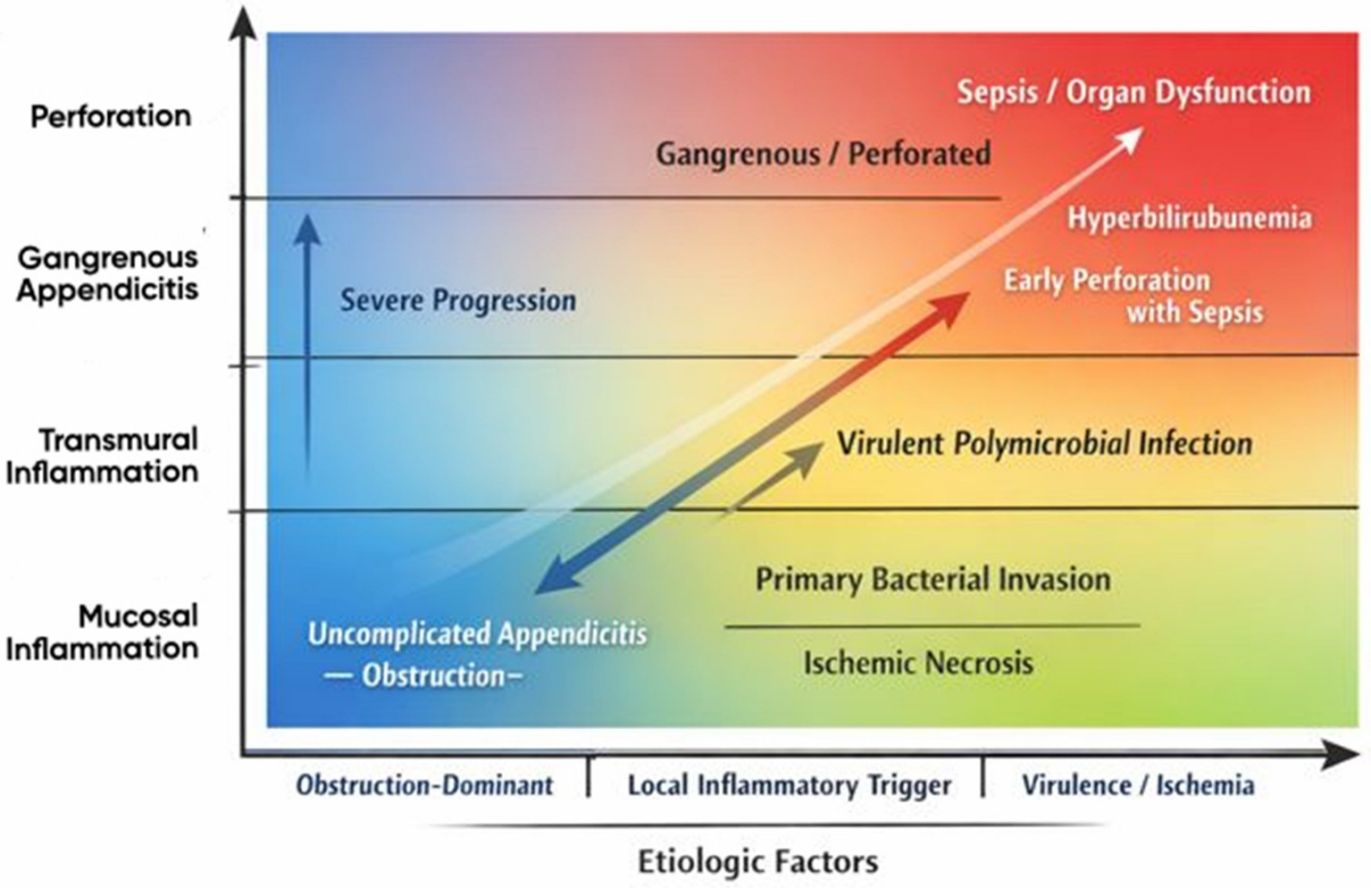
Hybrid severity–etiology framework of acute appendicitis. A schematic representation illustrating the conceptual separation between etiologic substrate and local pathological severity in acute appendicitis. The model demonstrates how different initiating biological mechanisms may lead to variable patterns of structural tissue injury and disease progression.

Figure 1 illustrates the conceptual separation between the etiologic substrate and local pathological severity within acute appendicitis. The model emphasizes that similar degrees of structural tissue injury may arise from different initiating biological mechanisms, while comparable etiologic contexts may progress toward markedly different pathological outcomes. This two-axis interpretation helps explain why appendicitis presentations frequently demonstrate discordance between symptom duration, microbiological findings, intraoperative appearance, and histopathological severity. By separating disease initiation from structural progression, the framework moves beyond a purely linear continuum model and provides a more flexible conceptual structure for interpreting heterogeneous appendicitis phenotypes.

### Host response as a third modulating dimension

While the hybrid severity–etiology framework reconciles many inconsistencies observed in acute appendicitis, it does not fully explain the marked interindividual variability in host systemic inflammatory and biochemical responses among patients with comparable local pathology and similar etiologic substrates. Accumulating clinical and biomarker data suggest that host-specific factors represent an additional, independent dimension modulating disease expression^[^9–13,20^]^.

Within the present framework, the term “host systemic response” refers collectively to the inflammatory, biochemical, physiological, and clinical manifestations generated by the host in response to appendiceal injury.

Host response in acute appendicitis encompasses the magnitude and character of the systemic inflammatory, immunologic, and metabolic reaction to a local appendiceal insult. This response is reflected in commonly used biomarkers, including C-reactive protein, procalcitonin, and serum bilirubin, as well as in clinical manifestations of systemic inflammatory response. Importantly, these markers do not directly measure the extent of local tissue injury or define the underlying etiologic mechanism; rather, they capture how the host responds to that injury^[^9–13^]^.

Recognition of host response as a distinct modulating dimension helps explain observations that remain difficult to reconcile within two-axis models. Histologically severe appendicitis, including transmural inflammation and localized peritonitis, may occur in patients with minimal or absent systemic biochemical derangement, suggesting a contained or tolerant host response phenotype^[^10–13,18^]^. Conversely, pronounced systemic inflammatory response may develop early in the disease course in some patients despite limited local pathological damage, reflecting an amplified host response rather than advanced local severity^[^9,11,20^]^.

This host-dependent variability also helps explain the inconsistent performance of biomarkers across studies. Biomarkers often demonstrate high specificity but limited sensitivity for complicated appendicitis because they primarily reflect systemic inflammatory amplification rather than local pathological severity^[^9–13,20^]^. Consequently, discordant biomarker findings may represent differences in host-response intensity rather than diagnostic failure.

Host response is influenced by a range of factors, including innate immune sensitivity, metabolic state, endotoxin handling, hepatic inflammatory signaling, comorbid conditions, immune phenotypes, and possibly genetic predisposition. These determinants act independently of, yet interact dynamically with, etiological drivers and local pathological processes. As a result, similar initiating mechanisms and comparable degrees of structural tissue injury may occupy markedly different positions along the host systemic response dimension.

This host-dependent variability aligns with emerging precision medicine paradigms, in which disease behavior is increasingly understood through biologically defined response patterns rather than anatomical severity alone. Within such frameworks, appendicitis may be conceptualized not only by its structural stage but also by its inflammatory endotype, characterized by distinct systemic amplification profiles. Recognizing these host-specific response patterns provides a biologically plausible explanation for heterogeneous biomarker performance and variable clinical trajectories observed across patients.

Incorporating host systemic response as an independent axis, therefore, transforms the hybrid framework into a fully multidimensional model. Etiologic substrate defines the initiating context, local pathological severity defines the extent of structural injury, and host response determines the degree of inflammatory amplification or containment. This structured separation enhances conceptual clarity and provides a biologically grounded basis for interpreting discordant clinicopathological findings.

**Figure 2.**
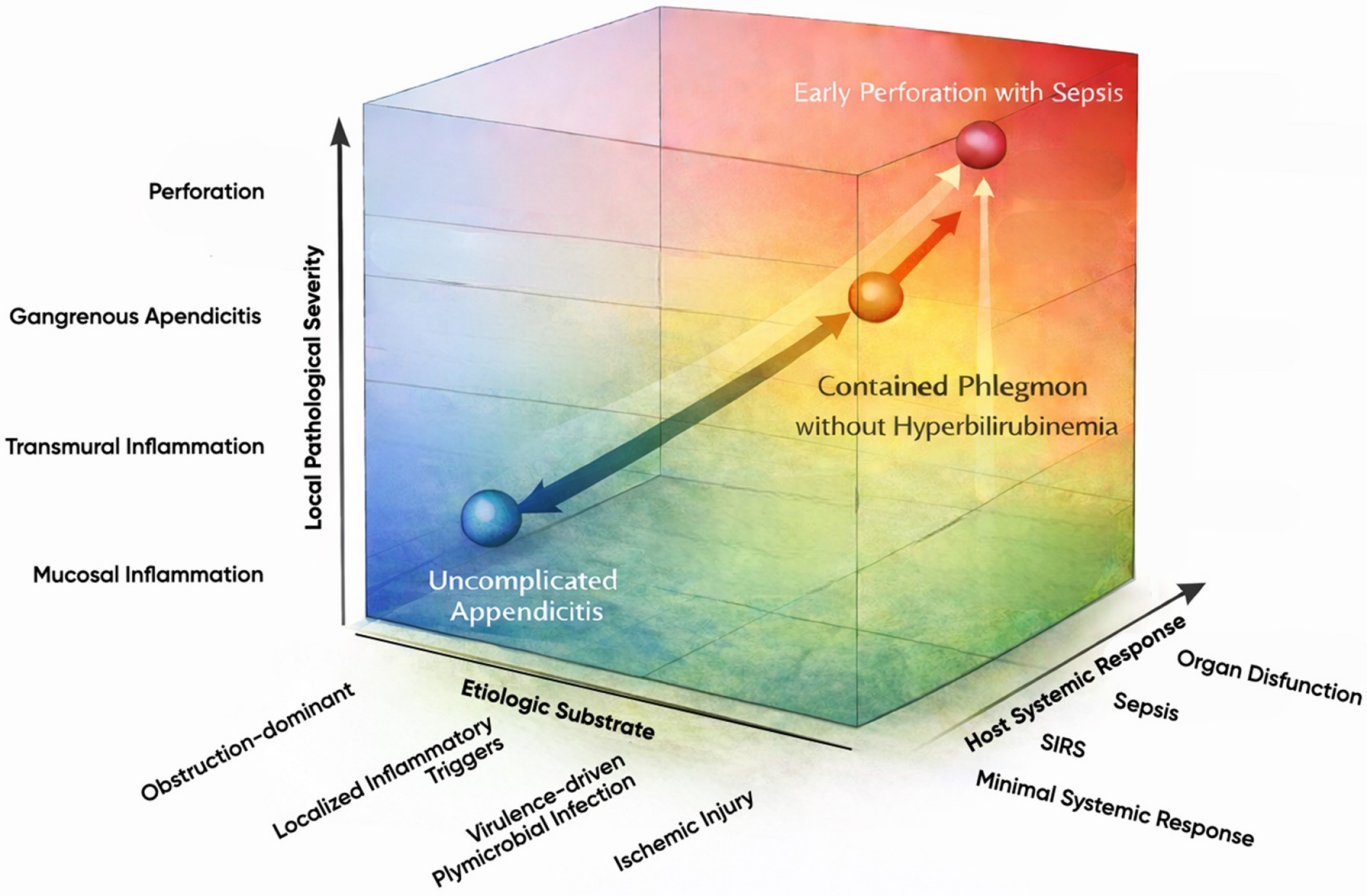
Three-dimensional framework integrating etiology, pathological severity, and host systemic response in acute appendicitis. A conceptual model illustrating the interaction between etiologic substrate, local pathological severity, and host systemic response as partially independent but biologically interconnected dimensions of appendicitis heterogeneity. The diagram demonstrates how similar pathological severity may be associated with variable systemic inflammatory and biomarker profiles. Two illustrative examples highlight relative axis independence: (1) severe local inflammation with limited systemic amplification (“contained phlegmon without hyperbilirubinemia”) and (2) early perforation associated with a pronounced systemic response (“early perforation with sepsis”). The model emphasizes that structural tissue injury and systemic inflammatory expression may vary disproportionately depending on the etiologic context and host-response characteristics.

Figure 2 extends the hybrid severity–etiology framework by incorporating the host systemic response as an independent third dimension modulating clinical disease expression. The model illustrates how patients with comparable local pathological severity may demonstrate markedly different inflammatory and biochemical profiles depending on host-response intensity, while similar biomarker patterns may arise from distinct etiologic and pathological contexts. This multidimensional interpretation provides a biologically plausible explanation for the inconsistent performance of inflammatory biomarkers across appendicitis studies and for the frequent dissociation observed between histopathological findings, systemic inflammatory expression, and clinical trajectory. By integrating etiologic substrate, structural tissue injury, and host-response amplification into a unified conceptual space, the framework offers a more comprehensive interpretative model for appendicitis heterogeneity.

### Operational interpretation of the multidimensional framework

For the proposed framework to be clinically and scientifically useful, each of its three dimensions must be interpretable through observable pathological, clinical, microbiological, and biochemical parameters. Although the present model is conceptual and not intended as a formal scoring system, each axis may be operationally approximated using currently available diagnostic information. Table 1 summarizes the three dimensions of the framework, together with representative clinical indicators that may assist in their practical interpretation.

**Table 1:** Summary of the three dimensions of acute appendicitis and their representative clinical indicators.

Dimension	Definition	Examples of clinical indicators
Etiologic substrate	Initiating biological mechanism	Fecalith, lymphoid hyperplasia, ischemia, endometriosis, diverticulum, microbiological profile
Local pathological severity	Extent of tissue injury	Catarrhal, phlegmonous, gangrenous, perforated appendicitis, abscess, peritonitis
Host systemic response	Magnitude of inflammatory amplification	CRP, NLR, bilirubin, procalcitonin, sepsis criteria, organ dysfunction

The etiologic substrate axis reflects the dominant biological mechanism initiating appendiceal inflammation. In clinical practice, this dimension may be inferred through a combination of radiological, intraoperative, microbiological, and histopathological findings. Obstruction-related processes, virulent polymicrobial infection, ischemic injury, and incidental pathological conditions may each represent distinct etiologic contexts influencing disease behavior and inflammatory progression.

The local pathological severity axis represents the anatomical extent of tissue injury and peritoneal involvement. This dimension may be assessed using histopathological examination, operative findings, and imaging-based severity assessments. Disease severity ranges from localized mucosal inflammation to transmural necrosis, perforation, abscess formation, and diffuse peritonitis. Existing operative grading systems and pathological classifications may, therefore, serve as practical tools for positioning patients along this structural severity axis.

The host systemic response axis reflects the magnitude of inflammatory amplification generated by the appendiceal insult. This dimension may be estimated using systemic inflammatory biomarkers, physiologic derangement, and clinical severity indices. Parameters such as C-reactive protein, neutrophil-to-lymphocyte ratio, procalcitonin, serum bilirubin, sepsis criteria, and markers of organ dysfunction may collectively reflect host-response intensity. Importantly, no single biomarker fully defines this axis, and biomarker expression should be interpreted as a composite reflection of host systemic inflammatory amplification rather than as a direct surrogate for local tissue destruction.

Operationally, the framework allows patients with similar pathological severity to occupy different positions along the host-response axis, while patients with comparable biomarker profiles may arise from distinct etiologic substrates. This structured separation may improve the interpretation of discordant clinicopathological findings and facilitate more biologically informed patient stratification in future studies.

### Relationship to existing classification and grading systems

The proposed multidimensional framework is not intended to replace existing operative, radiologic, or severity-based classification systems for acute appendicitis. Rather, it provides a broader interpretative structure within which these systems may be contextualized according to etiologic substrate, local pathological severity, and host systemic response.

Contemporary operative grading systems primarily characterize the anatomical extent of appendiceal and peritoneal involvement, including gangrene, perforation, abscess formation, and diffuse peritonitis^[^1,25^]^. Similarly, imaging-based classifications focus predominantly on structural disease severity identified through computed tomography or ultrasonography, while clinical trial stratification commonly dichotomizes patients into uncomplicated and complicated appendicitis groups^[^6,7,26^]^. These approaches remain highly valuable for surgical decision-making, treatment allocation, and outcome reporting.

However, most currently used classifications primarily describe local pathological severity and do not explicitly distinguish between initiating etiologic mechanisms and host-specific systemic inflammatory amplification. As a result, patients with similar anatomical disease severity may demonstrate markedly different biomarker profiles, microbiological characteristics, and clinical trajectories.

Within the proposed framework, existing grading systems may, therefore, be interpreted primarily as descriptors of the local pathological severity axis, while additional clinical, microbiological, and biochemical information contributes to the characterization of the etiologic substrate and host-response dimensions. In this context, the multidimensional model functions as an integrative interpretative framework capable of incorporating established classification systems rather than competing with them.

Such integration may improve clinicopathological correlation, reduce conceptual ambiguity in biomarker studies, and facilitate more biologically informed patient stratification in future appendicitis research.

## Implications for research design and biomarker interpretation

Adoption of a multidimensional framework has important implications for the design and interpretation of future appendicitis studies^[^1,7,8^]^. Much of the inconsistency in the literature may reflect conceptual ambiguity arising from the conflation of disease severity with disease etiology^[^2,4^]^.

Patient stratification should distinguish etiologic context, local pathological severity, and host systemic response^[^25,26^]^. Biomarker studies should clearly define whether their endpoints reflect structural tissue injury or systemic inflammatory amplification^[^9–13^]^. Within this framework, discordant biomarker findings should not be viewed as diagnostic failures but as manifestations of differing host-response intensity despite comparable pathological severity. More granular reporting of pathological findings and objective measures of systemic response may, therefore, improve biological interpretation and comparability across studies^[^6,7,18,19^]^.

## Clinical and pathological implications

Applying a hybrid severity–etiology framework to acute appendicitis has direct implications for both clinical practice and pathological evaluation^[^1,14,15^]^. Elevated inflammatory markers or hyperbilirubinemia should be understood primarily as reflections of the host's systemic response rather than direct evidence of perforation or specific etiologic mechanisms^[^9–13^]^.

Pathological examination plays a central role in operationalizing the hybrid framework. Detailed histopathological assessment defines the local disease substrate, identifies incidental or contributory etiologic factors, and clarifies the extent of tissue injury and peritoneal involvement^[^18,19^]^. Thorough sampling is particularly important in women of reproductive age and in cases with atypical clinical or biochemical profiles^[^23,24^]^.

## Future directions and unresolved questions

Despite extensive investigation, several fundamental questions regarding acute appendicitis remain unresolved^[^2,5,6,26^]^. In particular, the relative contributions and interactions of etiologic mechanisms, local pathological severity, and host systemic response have yet to be systematically disentangled. Future research should move beyond binary classification schemes and instead adopt study designs capable of capturing this multidimensional disease complexity.

Importantly, the proposed framework is empirically testable. Prospective studies may position patients along three axes: etiologic substrate, local pathological severity, and host systemic response. Mapping patients within this multidimensional space may help determine how these domains independently and interactively influence clinical outcomes, complications, and treatment response. The proposed axes should be interpreted as conceptual and operational domains rather than as completely biologically separable entities.

Validation of the model may be achieved by examining whether the separation of etiologic substrate, local pathological severity, and host systemic response improves predictive performance compared to conventional binary classifications. Multivariable modeling, correlation analyses, cluster analysis, latent class analysis, and dimensional reduction techniques may help determine whether these dimensions represent reproducible yet distinct domains of appendicitis biology. Comparative evaluation using outcome-based risk stratification and receiver operating characteristic analysis may further clarify the clinical utility of multidimensional classification.

Advanced methodologies, including immune phenotyping, cytokine profiling, transcriptomic analysis, and microbiome characterization, may further clarify host–pathogen interactions and identify reproducible endotypes within appendicitis presentations. Such approaches align with emerging precision medicine paradigms, in which disease behavior is understood not solely through anatomical severity but through biologically defined response patterns.

Clinically, operationalizing this framework may enhance decision-making by distinguishing patients with severe local pathology but limited systemic response from those exhibiting early systemic amplification despite modest structural injury. This distinction may influence perioperative management, antibiotic strategies, and monitoring intensity. Ultimately, systematic validation across multicenter cohorts would determine whether multidimensional classification improves reproducibility across studies and strengthens the prediction of adverse outcomes.

As biomarker research, microbiome science, and immunologic profiling evolve, the refinement of this multidimensional framework may enable more precise clinicopathological correlation and support the development of biologically informed management strategies in acute appendicitis.

## Conclusions

Acute appendicitis represents a biologically heterogeneous condition that cannot be fully explained by severity-based or etiology-based models in isolation. Integrating these perspectives within a structured, multidimensional framework allows clearer differentiation between three fundamental descriptors of the disease: etiologic substrate, local pathological severity, and host systemic response.

Distinguishing these partially independent but interacting dimensions provides a coherent explanation for the frequent dissociation observed between histopathological findings, systemic biomarker expression, microbiological profiles, and clinical trajectories. By separating structural tissue injury from inflammatory amplification, this framework reduces conceptual ambiguity and clarifies the interpretation of discordant clinical and biochemical findings.

Adoption of such a multidimensional approach may improve reproducibility across clinical studies, refine risk stratification strategies, and support more biologically informed management decisions. Systematic evaluation of etiologic context, extent of local pathology, and magnitude of systemic inflammatory response may enable more precise clinicopathological correlation and facilitate future advances in individualized care for patients with acute appendicitis.

## Data Availability

All data are available upon request from the authors.
